# Is PET/CT Useful in Detecting *Pneumocystis jirovecii* Colonization in Patients with Primary Lung Cancer?

**DOI:** 10.3390/cancers18203274

**Published:** 2026-10-11

**Authors:** Maciej Nowicki, Ignacy Ilski, Jan Żywica, Igor Szczurek, Magdalena Szydłowicz, Błażej Łukianowski, Mariusz Chabowski

**Affiliations:** 1 Department of Surgery, 4th Military Clinical Hospital, 50-981 Wroclaw, Poland; maciejnowicki@mp.pl; 2 Student Research Club No. 180, Faculty of Medicine, Wroclaw Medical University, 50-368 Wroclaw, Poland; ignacy.ilski@student.umw.edu.pl (I.I.); jan.zywica@student.umw.edu.pl (J.Ż.); igor.szczurek@student.umw.edu.pl (I.S.); 3 Department ofBiology and Medical Parasitology, Faculty of Medicine, Wroclaw Medical University, 50-368 Wroclaw, Poland; magdalena.szydlowicz@umw.edu.pl; 4 Division of Clinical Pathology, Department of Clinical and Experimental Pathology, Wroclaw Medical University, 50-368 Wroclaw, Poland; blazej.lukianowski@umw.edu.pl; 5 Department of Clinical Surgical Sciences, Wroclaw University of Science and Technology, 50-376 Wroclaw, Poland

**Keywords:** lung cancer, *Pneumocystis jirovecii*, staging, SUV-max, PET

## Abstract

**Background/Objectives:** *Pneumocystis jirovecii* colonization induces persistent low-grade pulmonary inflammation, which may alter fluorodeoxyglucose (FDG) uptake in positron emission tomography/computed tomography (PET/CT). We aimed to evaluate whether asymptomatic infection influences the maximum standardized uptake value (SUVmax) or clinicopathological parameters of primary lung cancer. **Methods:** In this prospective single-center observational study, 107 consecutive patients undergoing surgical resection for primary lung cancer were enrolled. Preoperative FDG PET/CT scans were obtained within four weeks prior to surgery. Intraoperative respiratory secretions and lung tissue samples were analyzed for *P. jirovecii*, using nested polymerase chain reaction (nested-PCR) targeting the mitochondrial large subunit rRNA gene. Propensity score matching (PSM) adjusting for age, sex, and body mass index was performed across 1:1, 1:2, and 1:3 matching models. **Results:** *Pneumocystis jirovecii* colonization was confirmed in 12 of 107 patients (11.21%). In the unadjusted cohort, no significant differences in primary lesion SUVmax were identified between positive and negative patients (10.38 ± 6.50 vs. 8.86 ± 6.58, *p* = 0.411), nor across matched models (1:1: *p* = 0.555; 1:2: *p* = 0.951; 1:3: *p* = 0.926). Following PSM, patients without colonization exhibited significantly larger maximum tumor diameters in both 1:2 (30.71 ± 19.23 vs. 18.25 ± 6.63 mm, *p* = 0.035) and 1:3 matching models (29.08 ± 17.47 vs. 18.25 ± 6.63 mm, *p* = 0.035). **Conclusions:** We did not find any association between *P. jirovecii* colonization and preoperative metabolic tumor staging on FDG PET/CT. However, asymptomatic carriage was associated with smaller primary tumor dimensions following propensity adjustment in 1:2 and 1:3 matching models, warranting further exploration of host–fungal interactions within the pulmonary tumor microenvironment.

## Simple Summary

Lung cancer is commonly evaluated before surgery by using metabolic scans such as positron emission tomography/computed tomography. However, pulmonary infections and inflammation may also result in increased metabolic activity, potentially confounding cancer staging. *Pneumocystis jirovecii* is a fungus that frequently colonizes the airways of patients without causing clinical symptoms. We investigated whether colonization by this fungus is associated with differences in tumor metabolic measurements or tumor size in 107 patients undergoing lung cancer surgery. No association was found between fungal colonization and metabolic activity in the primary tumor in any of the analyzed groups, including the primary analysis and the 1:1, 1:2 and 1:3 propensity score-matched analyses. However, colonized patients had significantly smaller tumors after adjustment for baseline characteristics in the 1:2 and 1:3 matching models, but not in the 1:1 model. Further studies are warranted to confirm this association and to determine whether *P. jirovecii* colonization may be associated with the tumor microenvironment and contribute to differences in tumor growth.

## 1. Introduction

Lung cancer is the most frequently diagnosed cancer worldwide [1]. According to 2026 projections, primary lung cancer is estimated to account for 229,410 new cases and 124,990 deaths in the United States alone, positioning this malignancy at the forefront of oncological mortality [2]. Lung cancer is responsible for 23.0% of all cancer-related deaths in men, and 14.1% in women [1]. Patient survival in primary lung cancer is predominantly dictated by the TNM classification, with increasing tumor size being associated with progressively poorer survival [3].

Fluorodeoxyglucose (FDG) PET/CT (positron emission tomography/computed tomography) is a sensitive imaging modality that quantifies tumor metabolic activity for cancer detection, staging, restaging, and treatment response assessment. Tracer uptake is expressed in SUV units, with the maximum value designated as SUVmax. A higher SUVmax value may suggest a neoplastic process. However, elevated FDG accumulation is a non-specific finding, occurring not only in neoplastic processes but also in granulation tissue, infections, and various inflammatory states [4].

Pulmonary infections are a common and serious complication in individuals with lung cancer. *Pneumocystis jirovecii* is an opportunistic fungal pathogen that is capable of causing life-threatening *Pneumocystis* pneumonia (PJP) in immunocompromised hosts, such as patients receiving immunosuppressive therapies. Increased susceptibility to infection may also result from invasive medical interventions that disrupt physiological and anatomical barriers [5,6,7,8,9,10]. While microscopic identification in bronchoalveolar lavage (BAL) or sputum remains the diagnostic gold standard, highly sensitive PCR assays frequently detect *P. jirovecii* DNA that indicates asymptomatic colonization rather than active infection [11,12].

Data from previous studies may indicate a potential role of *Pneumocystis jirovecii* in the course of chronic pulmonary diseases by inducing inflammatory changes with lung injury, as well as through interactions with other factors, including tobacco exposure and pathogenic bacteria [13]. Because this pathogen interacts with the lung microenvironment and induces inflammatory responses, it can potentially affect oncological imaging findings [14]. Inflammatory processes driven by *P. jirovecii* colonization may result in increased metabolic activity in pulmonary tissue, complicating the interpretation of tracer avidity.

To date, only one report has evaluated clinical outcomes in patients with *P. jirovecii* colonization, focusing on overall survival in primary lung cancer patients with *P. jirovecii* colonization compared to uncolonized counterparts [15]. Furthermore, mycological examinations are not routinely performed in thoracic surgery departments. These gaps in the literature provided the rationale for the present study. The direct impetus for conducting our research was *P. jirovecii* colonization research initiated by Dr. Szydłowicz at Wroclaw Medical University (UMW). We decided to pursue this research to expand the scarce literature addressing the clinical aspects of *P. jirovecii* colonization in patients with primary lung cancer.

The aim of this study was to compare tumor metabolic activity (as assessed by SUVmax values in PET-CT) between patients with primary lung cancer colonized by *Pneumocystis jirovecii* and those without colonization.

## 2. Materials and Methods

This is a prospective, single-center, observational study, conducted in the Department of Surgery, Fourth Military Teaching Hospital in Wroclaw, Poland. The protocol of the study was approved by the Ethics Committee of Wroclaw Medical University, Poland (no. KB 898/2022). All patients provided written informed consent to have their data used in the study. To minimize selection bias, all subsequent eligible patients were prospectively included during the study period. Due to this fact, no formal sample size calculation was performed. The patients were recruited in the time period between 27 February 2023 and 25 May 2026.

Patients were eligible for inclusion if they signed an informed consent form, underwent surgery for primary lung tumors in our institution, or had a preoperative PET/CT scan and an intraoperative collection of respiratory secretions. Exclusion criteria were: presence of metastatic lung tumors, non-neoplastic lesions, and lack of informed consent. Based on these criteria, 107 patients were included in the study (54 males, 53 females, mean age: 69.4).

The PET/CT scan was obtained as part of the routine diagnostic workup for lung neoplasm in each patient within 4 weeks prior to surgery. Only patients without signs of acute infection were eligible for the procedure. Until August 2025, the scans were performed using a Discovery IQ system (BGO + PMT detectors, 26 cm axial FOV, 16-row CT, clinically detectable spatial resolution of approximately 6–7 mm), GE HealthCare, Waukesha, WI, USA. Since October 2025, an Omni Legend system has been used (digital BGO detectors, 32 cm axial FOV, 64-row CT, clinically detectable spatial resolution of approximately 4–8 mm), GE HealthCare, Waukesha, WI, USA. PET/CT examinations were performed pre-operatively, whereas colonization by *P. jirovecii* was detected after the operation; therefore, PET/CT assessment and SUVmax measurements were performed blinded to the *P. jirovecii* status. The scans were interpreted by an experienced radiologist. The SUVmax was recorded for each patient from the PET/CT and included in a statistical analysis.

Biological specimens collected for the detection of *Pneumocystis jirovecii* were secretions (RS—bronchial secretions from lower respiratory tract) and lung tissue fragments, including neoplastic lung tissue (NPL) and those with no pathological alterations (normal adjacent tissue, NAT). Respiratory secretions were collected by an anesthesiologist through an intubation tube during the operative procedure. Lung tissue fragments were retrieved intraoperatively from each patient. Respiratory samples were frozen at −20 °C without preservatives, whereas NPL and NAT samples were placed in *RNAlater™* stabilization solution (Thermo Fisher Scientific, Carlsbad, CA, USA) prior to molecular examination. DNA extracted from all samples was used as a template for the detection of *Pneumocystis* using nested-PCR targeting a fragment of the mitochondrial large subunit rRNA (*mtLSU* rRNA) gene, according to previously described protocols [16,17]. Patient samples were tested with a nested polymerase chain reaction (PCR) with a Taq polymerase and primers targeting a partial sequence of the *P. jirovecii* mtLSU rRNA gene to detect *P. jirovecii* DNA. In addition, to evaluate samples for the presence of *Pneumocystis* developmental forms, a histopathological examination of the lung tissue was performed using standard staining procedures, including Giemsa and Grocott–Gomori methenamine silver (GMS) staining, and indirect immunofluorescence assays (MonoFluo *P. jirovecii* IFA Test Kit; Bio-Rad, Hercules, CA, USA) were applied to respiratory secretions (RS) [16].

The excised lung parenchyma underwent pathomorphological examination as well. Evaluated features of the tumors were: size, dimensions, histological type, differentiation, vascular invasion and presence of necrosis within the tumor tissue.

The study cohort was divided into two subgroups: patients diagnosed with *P. jirovecii* (n = 12) and patients without *P. jirovecii* (n = 95). Population size between the groups is unequal. This situation might lead to bias due to differences in terms of features like sex, age, and weight. Therefore, propensity score matching was applied, controlling for age, sex and BMI to form a control group. The inclusion of additional covariates could have substantially reduced the number of eligible matches; therefore, it was not executed. Nearest-neighbor matching was applied as the matching method, as it is the most standard approach. No caliper was employed, as this could have considerably reduced the number of successfully matched pairs. Instead, each individual from the study group was matched with the best available counterpart from the control group. Matching was performed without replacement; each individual in the control group could be matched to only one individual in the study group. Matching was executed across three variants of numerical ratios between the positive group and the control group (1:1, 1:2, and 1:3). These ratios were selected to examine the effect of increasing the number of control subjects matched to each study subject. While 1:1 matching provides the closest correspondence between the groups, the 1:2 and 1:3 approaches allow more control subjects to be included in the analysis and may therefore increase statistical efficiency. Comparing the results obtained with the three matching ratios enables the assessment of the consistency of the results across different matching ratios.

SUVmax obtained from PET/CT scans was our primary parameter of interest. Other variables used in statistical analysis included: age, sex, BMI, smoking history, tumor histological type, occurrence of necrosis within the tumor and TNM staging of the tumor.

The mean, standard deviation, median, quartiles and range of quantitative variables are shown. For qualitative variables, absolute and relative frequencies (N and %) were reported. Chi-squared test (with Yates correction for 2 × 2 tables) or Fisher’s exact test (in case of low expected values) were used for comparisons of qualitative variables between groups. The Mann–Whitney test was used for comparisons of quantitative variables between two groups. The significance level was set to 0.05. All the analyses were conducted in R software, version 4.6.0, with a MatchIt package.

## 3. Results

### 3.1. Primary Analysis

A total of 107 patients were included in the analysis, of whom 12 (11.21%) tested positive for *Pneumocystis jirovecii* (the positive study group), while 95 patients (88.79%) constituted the negative control group. Detailed demographic and clinical characteristics of the patients prior to matching are presented in Table 1. In the primary analysis, no significant differences were observed between the pathogen-positive group and the uninfected group across all evaluated parameters (*p* > 0.05).

Gender distribution was identical in the positive group (50.00% female, n = 6; 50.00% male, n = 6) and comparable in the negative group (49.47% female, n = 47; 50.53% male, n = 48; *p* = 1.000). The mean age of the patients was 71.42 ± 6.13 years in the *P. jirovecii* (+) group and 69.14 ± 7.14 years in the *P. jirovecii* (-) group (*p* = 0.432). The mean body mass index (BMI) was 27.1 ± 3.63 kg/m^2^ in positive patients and 26.05 ± 4.3 kg/m^2^ in negative patients (*p* = 0.314). Regarding the histopathological diagnosis, adenocarcinoma predominated in both groups, identified in 66.67% (n = 8) of patients in the positive group and 58.95% (n = 56) in the negative group, followed by squamous cell carcinoma—25.00% (n = 3) vs. 28.42% (n = 27), respectively (*p* = 1.000). Clinical staging according to the TNM classification also revealed no significant differences; the majority of patients in the positive group presented with T1 (41.67%, n = 5) or T2 (50.00%, n = 6; *p* = 0.214) descriptors, N0 status (91.67%, n = 11; *p* = 0.806), and the absence of distant metastases, M0 (91.67%, n = 11; *p* = 0.383).

In the evaluation of the tumor parameters, the mean maximum tumor size in the positive group was 18.25 ± 6.63 mm (median: 16.5 mm; range: 11–32 mm), whereas in the negative group, it was 25.8 ± 15.29 mm (median: 21 mm; range: 8–85 mm). This demonstrated a trend toward larger tumor sizes in negative patients, which did not reach significance (*p* = 0.087). The mean tumor volume was 23.16 ± 24.74 cm^3^ versus 90.94 ± 159.62 cm^3^, respectively (*p* = 0.193). Intratumoral necrosis was present in 25.00% (n = 3) of patients with the pathogen and in 32.63% (n = 31) of uninfected individuals (*p* = 0.747). The mean standardized uptake values (SUVs) were 10.38 ± 6.5 and 8.86 ± 6.58, respectively (*p* = 0.411). Smoking history analysis indicated that 50.00% (n = 6) of positive patients and 52.63% (n = 50) of negative patients were current smokers (*p* = 0.913), with a mean smoking burden of 40.08 ± 23.06 versus 34.34 ± 21.55 pack-years (*p* = 0.522).

Detailed demographic and clinical characteristics of the patients prior to matching are presented in Table 1.

### 3.2. The 1:1 Matching Model

In the first model, each of the 12 positive patients was matched with one negative patient exhibiting the closest demographic and clinical characteristics. Within the cohorts constructed in this manner, no significant differences were observed across any of the analyzed parameters (all *p*-values > 0.05). The groups were fully balanced regarding gender (50.00% female and 50.00% male in both cohorts). The mean ages were 71.42 ± 6.13 years and 72.08 ± 6.01 years, respectively (*p* = 0.559), while the mean BMIs were 27.10 ± 3.63 kg/m^2^ and 26.46 ± 4.57 kg/m^2^, respectively (*p* = 0.843). The mean maximum tumor size in the matched control group was 25.08 ± 14.95 mm (median: 22.5 mm), which, compared to 18.25 ± 6.63 mm (median: 16.5 mm) in the positive group, did not represent a significant difference (*p* = 0.235). Similarly, tumor volume (23.16 ± 24.74 cm^3^ vs. 63.40 ± 86.99 cm^3^; *p* = 0.378) and the percentage of necrosis (2.08 ± 3.96% vs. 2.08 ± 4.50%; *p* = 1.000) remained without significant differences.

The results of the 1:1 propensity score matching model, including detailed comparisons of demographic and clinical parameters between the groups, are summarized in Table 2.

### 3.3. The 1:2 Matching Model

Increasing the sample size of the control group by matching two negative patients to each positive patient (N = 24 in the control group) revealed significant structural differences regarding tumor morphological parameters.

Maximum tumor size was found to be significantly larger among patients that were free of *P. jirovecii* compared to those with confirmed presence of the pathogen. The mean maximum tumor size in the control group was 30.71 ± 19.23 mm (median: 26.5 mm; range: 10–85 mm), whereas in the positive group, it was 18.25 ± 6.63 mm (median: 16.5 mm; range: 11–32 mm), representing a significant difference (*p* = 0.035 *). In terms of tumor volume, a distinct trend toward higher values was noted in the negative group (139.23 ± 213.20 cm^3^, median: 50.89 cm^3^) than in the positive group (23.16 ± 24.74 cm^3^, median: 14.51 cm^3^), which approached the threshold of significance (*p* = 0.057). The remaining analyzed parameters demonstrated no significant differences (*p* > 0.05).

Data reflecting the structural differences and morphological parameters obtained through the 1:2 matching model are detailed in Table 3.

### 3.4. The 1:3 Matching Model

In the final matching model, in which three negative patients were assigned to each positive patient (N = 36 in the control group), the associations observed in the 1:2 model were fully confirmed.

The maximum tumor size was significantly larger in patients without the presence of *P. jirovecii*. In the control group, this value averaged 29.08 ± 17.47 mm (median: 25 mm; range: 10–85 mm) compared to 18.25 ± 6.63 mm (median: 16.5 mm; range: 11–32 mm) in the pathogen-positive group (*p* = 0.035 *). Tumor volume in the negative group (118.01 ± 183.21 cm^3^, median: 43.20 cm^3^) was higher than in the positive group (23.16 ± 24.74 cm^3^, median: 14.51 cm^3^), remaining on the verge of significance (*p* = 0.051). No significant differences were observed between the groups regarding the remaining clinical and demographic characteristics, such as the histopathological diagnosis distribution (*p* = 0.884), T descriptor (*p* = 0.211), N status (*p* = 0.808), M status (*p* = 0.441), the presence of necrosis (*p* = 0.497), SUV values (*p* = 0.926) and smoking burden expressed in pack-years (*p* = 0.901).

The comprehensive comparisons and outcomes for the final 1:3 matching model are compiled in Table 4.

## 4. Discussion

We found no significant differences in SUVmax between the asymptomatic groups of patients that were positive and negative for *P. jirovecii*, both in the primary analysis and after PSM. In the unmatched cohort (12 vs. 95 patients), no significant differences in the histological features of the primary lung tumors between the groups were observed. However, following PSM, patients negative for *P. jirovecii* had a significantly larger maximum tumor diameter than those who were positive, both in the 1:2 matching model (30.71 vs. 18.25 mm, *p* = 0.035) and in the 1:3 matching model (29.08 vs. 18.25 mm, *p* = 0.035).

As previously mentioned, FDG uptake is not specific for malignancy; active pulmonary infections can also cause increased FDG uptake. This result has been described in pulmonary tuberculosis, aspergillosis, bacterial pneumonia and other inflammatory and infectious conditions. For instance, active pulmonary tuberculosis [18] may demonstrate substantial FDG uptake, with considerable overlap between tuberculous and malignant lesions, limiting the ability of SUV measurement alone to differentiate between these two. Similarly, aspergillosis may present with increased FDG uptake, especially in invasive aspergillosis [19]. Bacterial pneumonia can also result in visible FDG accumulation, often reaching SUVmax values that are comparable to those observed in primary lung cancers [20]. These studies illustrate that active pulmonary infections may influence FDG PET/CT interpretation through the inflammatory response they induce.

Only a limited number of studies have evaluated the role of PET/CT in the diagnosis of *Pneumocystis jirovecii* infections. Current guidelines identify high-resolution computed tomography as the most reliable radiological modality for the detection of PJP, whereas FDG PET/CT is considered an adjunctive imaging modality, which is especially useful in the early diagnosis of PJP in patients with normal chest radiographs [21]. The PET/CT findings correlate well with conventional imaging. However, the potential role of PET/CT in detecting *Pneumocystis jirovecii* colonization in patients with primary lung cancer has not been systematically investigated.

Previous studies have described PET/CT findings in clinically overt PJP; however, our study consisted of asymptomatic patients with *P. jirovecii* colonization. As highlighted by Morris and Norris, colonization is characterized by the presence of fungal organisms without clinical or radiological evidence of infection and is associated with a substantially lower fungal burden and a milder host inflammatory response than active PJP [22]. Consequently, colonization is unlikely to induce the degree of inflammatory activity required to substantially increase FDG uptake, which may explain the absence of differences in SUVmax values observed in our cohort.

Interestingly, while metabolic activity remained unchanged throughout all analyses, PSM 1:2 and 1:3 matching models revealed that *P. jirovecii*-colonized patients had significantly smaller tumors than non-colonized ones. This association became apparent after balancing baseline demographic characteristics between groups, suggesting that differences in age, sex, and body mass index may have partially weakened this relationship in the unmatched cohort. However, given the small number of colonized patients, this result should be interpreted cautiously and considered hypothesis-generating rather than confirmatory.

A possible biological explanation is that colonization may be favored by earlier stages of lung cancer, when the pulmonary architecture and local tissue microenvironment remain relatively preserved. The observed association may indicate that as the tumor grows and progresses, alterations in tissue architecture, vascularization, oxygenation, extracellular matrix and local immune conditions may occur. The resulting changes in the tissue environment may be less permissive to *P. jirovecii* persistence [16,23]. In patients with smaller and potentially less advanced tumors, relatively preserved alveolar architecture and surrounding pulmonary tissue may provide a more permissive niche for *P. jirovecii* colonization. Conversely, progressive tumor growth and associated tissue remodeling may alter or disrupt this niche, potentially reducing the ability of *P. jirovecii* to persist in the affected lung. Thus, the higher frequency of *P. jirovecii* colonization observed among patients with smaller tumors may represent a consequence of differences in the local pulmonary environment associated with tumor progression, rather than evidence that *P. jirovecii* itself limits tumor growth, although this hypothesis requires further confirmation.

A recent study by Szydłowicz et al. has identified *P. jirovecii* DNA not only in RS but also within the lung tumor tissue and adjacent non-neoplastic parenchyma [16]. Notably, tumor tissue did not appear to be a preferential site of *Pneumocystis* detection. In all cases in which the organism was detected in tumor tissue, it was also identified in the corresponding adjacent non-neoplastic tissue and respiratory secretions, suggesting that its presence within the tumor may reflect the broader distribution of *Pneumocystis* within the pulmonary environment rather than preferential localization to malignant tissue. Moreover, the previously reported focal and heterogeneous distribution of *P. jirovecii* within the lung further supports the potential importance of local tissue conditions in determining its detectability and persistence [24]. Collectively, those findings suggest that preservation of the pulmonary microenvironment may favor *P. jirovecii* colonization.

An important methodologic strength of the present study is its prospective design combined with standardized pre-operative PET-CT imaging and microbiological intra-operative sampling. All patients in our cohort underwent the procedures according to the same institutional protocol before pulmonary resection, thereby reducing potential selection bias. Another major strength of the study is the application of the PSM. By constructing matched cohorts, we sought to improve comparability between study groups while preserving the observational nature of the investigation. A further strength of the study is its multidisciplinary approach, combining expertise from thoracic surgery, parasitology and pathology.

This study has several limitations. First, it was a single-center study including only twelve patients with confirmed *P. jirovecii* colonization. The limited number of positive cases reduces the statistical power of the study and limits its ability to assess potential associations with clinical and pathological characteristics. Furthermore, we grouped patients in whom *P. jirovecii* was detected in adjacent non-neoplastic tissue, tumor tissue, or RS into a single colonized group. Dividing the patients according to the site of detection would have resulted in subgroups that were too small for meaningful statistical analysis.

Second, the PCR-based approach used for *P. jirovecii* detection identifies fungal DNA irrespective of organism viability and therefore cannot distinguish colonization from infection. However, in the absence of clinical and radiological features suggestive of PJP, patients with positive PCR results were interpreted as being colonized. Nevertheless, this methodological limitation should be considered when interpreting the findings, and further studies using complementary approaches to assess organism viability and burden are warranted.

## 5. Conclusions

In summary, in the present prospective study, we found no association between *P. jirovecii* colonization and the metabolic activity of primary lung tumors according to the preoperative FDG PET/CT SUVmax criteria. Subclinical fungal infection may therefore not induce sufficient local inflammation that could interfere with the interpretation of PET results during oncological staging. However, it should be noted that the use of propensity score matching showed the presence of a significant reverse association between *P. jirovecii* positivity and maximum tumor size in 1:2 and 1:3 matching models. Although these findings do not establish a causal relationship, they suggest that interactions between *P. jirovecii*, the pulmonary tumor microenvironment, and lung cancer biology may be more complex than previously recognized. Future large-scale multicenter prospective cohort studies should clarify the biomolecular basis of this phenomenon.

## Figures and Tables

**Table 1:** Primary analysis.

Parameter		*P. jirovecii*		*p*
		Positive (N = 12)	Negative (N = 95)	
Sex	Female	6 (50.00%)	47 (49.47%)	*p* = 1
	Male	6 (50.00%)	48 (50.53%)	
Age [years]	Mean (SD)	71.42 (6.13)	69.14 (7.14)	*p* = 0.432
	Median (quartiles)	70.5 (66.75–74.25)	70 (65–74)	
	Range	65–86	44–82	
	n	12	95	
BMI [kg/m^2^]	Mean (SD)	27.1 (3.63)	26.05 (4.3)	*p* = 0.314
	Median (quartiles)	27.16 (24.73–29.52)	25.71 (23.58–28.36)	
	Range	22.2–34.26	16.02–40.16	
	n	12	95	
Diagnosis	Adenocarcinoma	8 (66.67%)	56 (58.95%)	*p* = 1
	Squamous cell carcinoma	3 (25.00%)	27 (28.42%)	
	Other	1 (8.33%)	12 (12.63%)	
T staging	T1	5 (41.67%)	53 (55.79%)	*p* = 0.214
	T2	6 (50.00%)	28 (29.47%)	
	T3	0 (0.00%)	11 (11.58%)	
	T4	1 (8.33%)	3 (3.16%)	
N staging	N0	11 (91.67%)	76 (80.00%)	*p* = 0.806
	N1	1 (8.33%)	15 (15.79%)	
	N2	0 (0.00%)	4 (4.21%)	
M staging	M0	11 (91.67%)	92 (96.84%)	*p* = 0.383
	M1	1 (8.33%)	3 (3.16%)	
Max. tumor size [mm]	Mean (SD)	18.25 (6.63)	25.8 (15.29)	*p* = 0.087
	Median (quartiles)	16.5 (14.5–20)	21 (15–30.75)	
	Range	11–32	8–85	
	n	12	92	
Tumor volume [cm^3^]	Mean (SD)	23.16 (24.74)	90.94 (159.62)	*p* = 0.193
	Median (quartiles)	14.51 (9.07–26.7)	24.96 (8.08–84.19)	
	Range	4.61–93.83	0.84–801.11	
	n	12	92	
Necrosis within the tumor [%]	Mean (SD)	2.08 (3.96)	7.19 (18.05)	*p* = 0.528
	Median (quartiles)	0 (0–1.25)	0 (0–5)	
	Range	0–10	0–90	
	n	12	93	
Necrosis within the tumor	None	9 (75.00%)	62 (65.26%)	*p* = 0.747
	Present	3 (25.00%)	31 (32.63%)	
	Unknown	0 (0.00%)	2 (2.11%)	
Pleural invasion	PL0	6 (50.00%)	66 (69.47%)	*p* = 0.164
	PL1	4 (33.33%)	24 (25.26%)	
	PL2	2 (16.67%)	3 (3.16%)	
	PL3	0 (0.00%)	1 (1.05%)	
	Unknown	0 (0.00%)	1 (1.05%)	
SUV	Mean (SD)	10.38 (6.5)	8.86 (6.58)	*p* = 0.411
	Median (quartiles)	9.9 (5–14.2)	7.75 (4.38–11.57)	
	Range	3.1–21	0.7–44.2	
	n	11	94	
Smoking status	Never smoker	1 (8.33%)	13 (13.68%)	*p* = 0.913
	Ex-smoker	5 (41.67%)	32 (33.68%)	
	Current smoker	6 (50.00%)	50 (52.63%)	
Smoking [pack-years]	Mean (SD)	40.08 (23.06)	34.34 (21.55)	*p* = 0.522
	Median (quartiles)	40 (30–43.25)	40 (22–45)	
	Range	0–100	0–135	
	n	12	95	

*p*—Qualitative variables: chi-squared or Fisher’s exact test. Quantitative variables: Mann–Whitney test.

**Table 2:** Propensity score matching 1:1.

Parameter		*P. jirovecii*		*p*
		Positive (N = 12)	Negative (N = 12)	
Sex	Female	6 (50.00%)	6 (50.00%)	*p* = 1
	Male	6 (50.00%)	6 (50.00%)	
Age [years]	Mean (SD)	71.42 (6.13)	72.08 (6.01)	*p* = 0.559
	Median (quartiles)	70.5 (66.75–74.25)	73 (69.75–75)	
	Range	65–86	62–82	
	n	12	12	
BMI [kg/m^2^]	Mean (SD)	27.1 (3.63)	26.46 (4.57)	*p* = 0.843
	Median (quartiles)	27.16 (24.73–29.52)	27.16 (23.5–29.61)	
	Range	22.2–34.26	19.14–33.87	
	n	12	12	
Diagnosis	Adenocarcinoma	8 (66.67%)	6 (50.00%)	*p* = 0.733
	Squamous cell carcinoma	3 (25.00%)	4 (33.33%)	
	Other	1 (8.33%)	2 (16.67%)	
T staging	T1	5 (41.67%)	5 (41.67%)	*p* = 0.582
	T2	6 (50.00%)	5 (41.67%)	
	T3	0 (0.00%)	2 (16.67%)	
	T4	1 (8.33%)	0 (0.00%)	
N staging	N0	11 (91.67%)	10 (83.33%)	*p* = 1
	N1	1 (8.33%)	2 (16.67%)	
	N2	0 (0.00%)	0 (0.00%)	
M staging	M0	11 (91.67%)	12 (100.00%)	*p* = 1
	M1	1 (8.33%)	0 (0.00%)	
Max. tumor size [mm]	Mean (SD)	18.25 (6.63)	25.08 (14.95)	*p* = 0.235
	Median (quartiles)	16.5 (14.5–20)	22.5 (16.75–29.25)	
	Range	11–32	10–65	
	n	12	12	
Tumor volume [cm^3^]	Mean (SD)	23.16 (24.74)	63.4 (86.99)	*p* = 0.378
	Median (quartiles)	14.51 (9.07–26.7)	31.37 (7.31–69.35)	
	Range	4.61–93.83	3.39–299.5	
	n	12	12	
Necrosis within the tumor [%]	Mean (SD)	2.08 (3.96)	2.08 (4.5)	*p* = 1
	Median (quartiles)	0 (0–1.25)	0 (0–1.25)	
	Range	0–10	0–15	
	n	12	12	
Necrosis within the tumor	None	9 (75.00%)	9 (75.00%)	*p* = 1
	Present	3 (25.00%)	3 (25.00%)	
Pleural invasion	PL0	6 (50.00%)	8 (66.67%)	*p* = 0.574
	PL1	4 (33.33%)	4 (33.33%)	
	PL2	2 (16.67%)	0 (0.00%)	
	PL3	0 (0.00%)	0 (0.00%)	
SUV	Mean (SD)	10.38 (6.5)	8.96 (2.86)	*p* = 0.555
	Median (quartiles)	9.9 (5–14.2)	9.4 (7.78–11.6)	
	Range	3.1–21	2.7–11.9	
	n	11	12	
Smoking status	Never smoker	1 (8.33%)	3 (25.00%)	*p* = 0.496
	Ex-smoker	5 (41.67%)	3 (25.00%)	
	Current smoker	6 (50.00%)	6 (50.00%)	
Smoking [pack-years]	Mean (SD)	40.08 (23.06)	31.67 (21.25)	*p* = 0.742
	Median (quartiles)	40 (30–43.25)	40 (15–42.5)	
	Range	0–100	0–60	
	n	12	12	

*p*—Qualitative variables: Chi-squared or Fisher’s exact test. Quantitative variables: Mann–Whitney test.

**Table 3:** Propensity score matching 1:2.

Parameter		*P. jirovecii*		*p*
		Positive (N = 12)	Negative (N = 24)	
Sex	Female	6 (50.00%)	12 (50.00%)	*p* = 1
	Male	6 (50.00%)	12 (50.00%)	
Age [years]	Mean (SD)	71.42 (6.13)	72.42 (5.36)	*p* = 0.449
	Median (quartiles)	70.5 (66.75–74.25)	73.5 (69.75–75.25)	
	Range	65–86	62–82	
	n	12	24	
BMI [kg/m^2^]	Mean (SD)	27.1 (3.63)	26.15 (3.97)	*p* = 0.497
	Median (quartiles)	27.16 (24.73–29.52)	25.71 (23.74–28.52)	
	Range	22.2–34.26	19.14–34.71	
	n	12	24	
Diagnosis	Adenocarcinoma	8 (66.67%)	14 (58.33%)	*p* = 1
	Squamous cell carcinoma	3 (25.00%)	7 (29.17%)	
	Other	1 (8.33%)	3 (12.50%)	
T staging	T1	5 (41.67%)	12 (50.00%)	*p* = 0.379
	T2	6 (50.00%)	7 (29.17%)	
	T3	0 (0.00%)	4 (16.67%)	
	T4	1 (8.33%)	1 (4.17%)	
N staging	N0	11 (91.67%)	19 (79.17%)	*p* = 0.76
	N1	1 (8.33%)	4 (16.67%)	
	N2	0 (0.00%)	1 (4.17%)	
M staging	M0	11 (91.67%)	23 (95.83%)	*p* = 1
	M1	1 (8.33%)	1 (4.17%)	
Max. tumor size [mm]	Mean (SD)	18.25 (6.63)	30.71 (19.23)	*p* = 0.035 *
	Median (quartiles)	16.5 (14.5–20)	26.5 (16.75–35.25)	
	Range	11–32	10–85	
	n	12	24	
Tumor volume [cm^3^]	Mean (SD)	23.16 (24.74)	139.23 (213.2)	*p* = 0.057
	Median (quartiles)	14.51 (9.07–26.7)	50.89 (11.46–147.06)	
	Range	4.61–93.83	3.39–801.11	
	n	12	24	
Necrosis within the tumor [%]	Mean (SD)	2.08 (3.96)	10 (20.22)	*p* = 0.355
	Median (quartiles)	0 (0–1.25)	0 (0–6.25)	
	Range	0–10	0–70	
	n	12	24	
Necrosis within the tumor	None	9 (75.00%)	15 (62.50%)	*p* = 0.709
	Present	3 (25.00%)	9 (37.50%)	
Pleural invasion	PL0	6 (50.00%)	16 (66.67%)	*p* = 0.187
	PL1	4 (33.33%)	7 (29.17%)	
	PL2	2 (16.67%)	0 (0.00%)	
	PL3	0 (0.00%)	1 (4.17%)	
SUV	Mean (SD)	10.38 (6.5)	10.4 (5.01)	*p* = 0.951
	Median (quartiles)	9.9 (5–14.2)	9.6 (7.18–12.08)	
	Range	3.1–21	2.7–26	
	n	11	24	
Smoking status	Never smoker	1 (8.33%)	3 (12.50%)	*p* = 0.885
	Ex-smoker	5 (41.67%)	8 (33.33%)	
	Current smoker	6 (50.00%)	13 (54.17%)	
Smoking [pack-years]	Mean (SD)	40.08 (23.06)	35.38 (20.93)	*p* = 0.848
	Median (quartiles)	40 (30–43.25)	40 (23.75–50)	
	Range	0–100	0–80	
	n	12	24	

*p*—Qualitative variables: Chi-squared or Fisher’s exact test. Quantitative variables: Mann–Whitney test. * Statistically significant (*p* < 0.05).

**Table 4:** Propensity score matching 1:3.

Parameter		*P. jirovecii*		*p*
		Positive (N = 12)	Negative (N = 36)	
Sex	Female	6 (50.00%)	18 (50.00%)	*p* = 1
	Male	6 (50.00%)	18 (50.00%)	
Age [years]	Mean (SD)	71.42 (6.13)	72.25 (5.38)	*p* = 0.385
	Median (quartiles)	70.5 (66.75–74.25)	74 (69–75.25)	
	Range	65–86	59–82	
	n	12	36	
BMI [kg/m^2^]	Mean (SD)	27.1 (3.63)	25.98 (3.55)	*p* = 0.349
	Median (quartiles)	27.16 (24.73–29.52)	25.95 (23.74–27.9)	
	Range	22.2–34.26	19.14–34.71	
	n	12	36	
Diagnosis	Adenocarcinoma	8 (66.67%)	20 (55.56%)	*p* = 0.884
	Squamous cell carcinoma	3 (25.00%)	12 (33.33%)	
	Other	1 (8.33%)	4 (11.11%)	
T staging	T1	5 (41.67%)	20 (55.56%)	*p* = 0.211
	T2	6 (50.00%)	10 (27.78%)	
	T3	0 (0.00%)	5 (13.89%)	
	T4	1 (8.33%)	1 (2.78%)	
N staging	N0	11 (91.67%)	28 (77.78%)	*p* = 0.808
	N1	1 (8.33%)	6 (16.67%)	
	N2	0 (0.00%)	2 (5.56%)	
M staging	M0	11 (91.67%)	35 (97.22%)	*p* = 0.441
	M1	1 (8.33%)	1 (2.78%)	
Max. tumor size [mm]	Mean (SD)	18.25 (6.63)	29.08 (17.47)	*p* = 0.035 *
	Median (quartiles)	16.5 (14.5–20)	25 (16.75–35.25)	
	Range	11–32	10–85	
	n	12	36	
Tumor volume [cm^3^]	Mean (SD)	23.16 (24.74)	118.01 (183.21)	*p* = 0.051
	Median (quartiles)	14.51 (9.07–26.7)	43.2 (11.46–147.06)	
	Range	4.61–93.83	3.39–801.11	
	n	12	36	
Necrosis within the tumor [%]	Mean (SD)	2.08 (3.96)	9.03 (19)	*p* = 0.348
	Median (quartiles)	0 (0–1.25)	0 (0–5)	
	Range	0–10	0–70	
	n	12	36	
Necrosis within the tumor	None	9 (75.00%)	22 (61.11%)	*p* = 0.497
	Present	3 (25.00%)	14 (38.89%)	
Pleural invasion	PL0	6 (50.00%)	24 (66.67%)	*p* = 0.089
	PL1	4 (33.33%)	11 (30.56%)	
	PL2	2 (16.67%)	0 (0.00%)	
	PL3	0 (0.00%)	1 (2.78%)	
SUV	Mean (SD)	10.38 (6.5)	11.12 (7.71)	*p* = 0.926
	Median (quartiles)	9.9 (5–14.2)	9.65 (6.35–12.62)	
	Range	3.1–21	1.4–44.2	
	n	11	36	
Smoking status	Never smoker	1 (8.33%)	5 (13.89%)	*p* = 0.635
	Ex-smoker	5 (41.67%)	10 (27.78%)	
	Current smoker	6 (50.00%)	21 (58.33%)	
Smoking [pack-years]	Mean (SD)	40.08 (23.06)	36.22 (21.78)	*p* = 0.901
	Median (quartiles)	40 (30–43.25)	40 (25–50)	
	Range	0–100	0–90	
	n	12	36	

*p*—Qualitative variables: Chi-squared or Fisher’s exact test. Quantitative variables: Mann–Whitney test. * Statistically significant (*p* < 0.05).

## Data Availability

The data presented in this study are available on request from the corresponding author.

## Abbreviations

The following abbreviations are used in this manuscript:

FDG	fluorodeoxyglucose
PET/CT	positron emission tomography/computed tomography
PCR	polymerase chain reaction
PSM	propensity score matching
SUV	standardized uptake value
PJP	*Pneumocystis jirovecii* pneumonia
BAL	bronchoalveolar lavage
DNA	deoxyribonucleic acid
BMI	body mass index
SD	standard deviation
RS	respiratory secretions
NPL	neoplastic tissue
NAT	normal adjacent tissue
GMS	Grocott–Gomori methenamine silver

## References

[1] Sung H., Filho A.M., Laversanne M., Ferlay J., Siegel R.L., Soerjomataram I., Jemal A., Bray F. (2026). Global Cancer Statistics 2024: GLOBOCAN Estimates of Incidence and Mortality Worldwide for 34 Cancers in 186 Countries. CA Cancer J. Clin..

[2] Siegel R.L., Kratzer T.B., Wagle N.S., Sung H., Jemal A. (2026). Cancer Statistics, 2026. CA Cancer J. Clin..

[3] Van Schil P.E., Asamura H., Nishimura K.K., Rami-Porta R., Kim Y.T., Bertoglio P., Cangir A.K., Donington J., Fang W., Giroux D.J. (2024). The International Association for the Study of Lung Cancer Lung Cancer Staging Project: Proposals for the Revisions of the T-Descriptors in the Forthcoming Ninth Edition of the TNM Classification for Lung Cancer. J. Thorac. Oncol..

[4] Boellaard R., Delgado-Bolton R., Oyen W.J.G., Giammarile F., Tatsch K., Eschner W., Verzijlbergen F.J., Barrington S.F., Pike L.C., Weber W.A. (2015). FDG PET/CT: EANM Procedure Guidelines for Tumour Imaging: Version 2.0. Eur. J. Nucl. Med. Mol. Imaging.

[5] Wang Y., Zheng C., Sun Y., Zhang Y. (2026). Immune Responses to Pneumocystis in HIV-Infected and Non-HIV Immunocompromised Hosts. J. Inflamm. Res..

[6] Togashi Y., Masago K., Ito Y., Sakamori Y., Okuda C., Fukuhara A., Nagai H., Kim Y.H., Mishima M. (2013). *Pneumocystis jiroveci* Pneumonia and Colonization in Patients with Advanced Lung Cancer. Oncol. Lett..

[7] Lee Y.-T., Chuang M.-L. (2018). *Pneumocystis jirovecii* Pneumonia in AIDS and Non-AIDS Immunocompromised Patients—An Update. J. Infect. Dev. Ctries..

[8] Zhang X., Jiang N., Wang L., Liu H., He R. (2017). Chronic Obstructive Pulmonary Disease and Risk of Lung Cancer: A Meta-Analysis of Prospective Cohort Studies. Oncotarget.

[9] Sarihan S., Ercan I., Saran A., Cetintas S.K., Akalin H., Engin K. (2005). Evaluation of Infections in Non-Small Cell Lung Cancer Patients Treated with Radiotherapy. Cancer Detect. Prev..

[10] Vento S., Cainelli F., Temesgen Z. (2008). Lung Infections after Cancer Chemotherapy. Lancet Oncol..

[11] White P.L., Backx M., Barnes R.A. (2017). Diagnosis and Management of *Pneumocystis jirovecii* Infection. Expert Rev. Anti Infect. Ther..

[12] Jaramillo Cartagena A., Asowata O.E., Ng D., Babady N.E. (2025). An Overview of the Laboratory Diagnosis of *Pneumocystis jirovecii* Pneumonia. J. Clin. Microbiol..

[13] Gutiérrez S., Respaldiza N., Campano E., Martínez-Risquez M.T., Calderón E.J., De La Horra C. (2011). *Pneumocystis jirovecii* Colonization in Chronic Pulmonary Disease. Parasite.

[14] Rojas D.A., Ponce C.A., Bustos A., Cortés V., Olivares D., Vargas S.L. (2023). Pneumocystis Exacerbates Inflammation and Mucus Hypersecretion in a Murine, Elastase-Induced-COPD Model. J. Fungi.

[15] Zaini J., A’la Al Maududi A., Fillahihasanah T., Rizqy Fadhillah M., Pradono P., Haryanto B., Adawiyah R., Setianingrum F., Rozaliyani A., Syahruddin E. (2022). *Pneumocystis jirovecii* Colonization in Bronchoalveolar Lavage among Naïve Non-Small Cell Lung Cancer from Tertiary Respiratory Hospital in Jakarta, Indonesia. J. Infect. Dev. Ctries..

[16] Szydłowicz M., Zajączkowska Ż., Chabowski M., Nowicki M., Łukianowski B., Gajdzis P., Kváč M., Calderón E.J., Le Gal S., Kicia M. (2026). Multi-Site Screening for *Pneumocystis jirovecii* in Lung Cancer: Possible Tumour Tissue Colonization. Front. Cell. Infect. Microbiol..

[17] Wakefield A.E. (1996). DNA Sequences Identical to *Pneumocystis carinii* f. sp. *carinii* and *Pneumocystis carinii* f. sp. *hominis* in Samples of Air Spora. J. Clin. Microbiol..

[18] Santos A.P., Souza Rodrigues R., Aarntzen E.H.J.G., Rosado de Castro P.H., Magis-Escurra C., de Queiroz Mello F.C. (2025). 18F-Fluorodeoxyglucose PET/CT Scans for Thoracic Tuberculosis: Current Evidence and Future Perspectives. Eur. Respir. Rev..

[19] Kim J.Y., Yoo J.-W., Oh M., Park S.H., Shim T.S., Choi Y.Y., Ryu J.-S. (2013). ^18^F-Fluoro-2-Deoxy-D-Glucose Positron Emission Tomography/Computed Tomography Findings Are Different between Invasive and Noninvasive Pulmonary Aspergillosis. J. Comput. Assist. Tomogr..

[20] Rahman W.T., Wale D.J., Viglianti B.L., Townsend D.M., Manganaro M.S., Gross M.D., Wong K.K., Rubello D. (2019). The Impact of Infection and Inflammation in Oncologic ^18^F-FDG PET/CT Imaging. Biomed. Pharmacother. Biomed. Pharmacother..

[21] Cooley L., Dendle C., Wolf J., Teh B.W., Chen S.C., Boutlis C., Thursky K.A. (2014). Consensus Guidelines for Diagnosis, Prophylaxis and Management of *Pneumocystis jirovecii* Pneumonia in Patients with Haematological and Solid Malignancies, 2014. Intern. Med. J..

[22] Morris A., Norris K.A. (2012). Colonization by *Pneumocystis jirovecii* and Its Role in Disease. Clin. Microbiol. Rev..

[23] Huynh M., Crane M.J., Jamieson A.M. (2023). The Lung, the Niche, and the Microbe: Exploring the Lung Microbiome in Cancer and Immunity. Front. Immunol..

[24] Vargas S.L., Ponce C., Bustamante R., Calderón E., Nevez G., De Armas Y., Matos O., Miller R.F., Gallo M.J. (2017). Importance of Tissue Sampling, Laboratory Methods, and Patient Characteristics for Detection of Pneumocystis in Autopsied Lungs of Non-Immunosuppressed Individuals. Eur. J. Clin. Microbiol. Infect. Dis..

